# Utilization Pattern and Related Knowledge of Nasal Decongestants Among the General Population in the Al-Qassim Region, Saudi Arabia

**DOI:** 10.7759/cureus.88923

**Published:** 2025-07-28

**Authors:** Mohammed A Altatan, Abdulaziz H Alluhayb, Faisal E Alessa, Saud K Alamri, Mohammed A Alfallaj, Omar F Almansour, Waleed A Alhazmi

**Affiliations:** 1 College of Medicine, Qassim University, Buraidah, SAU; 2 Department of Otolaryngology, Head and Neck Surgery, Qassim University, Buraidah, SAU

**Keywords:** knowledge assessment, nasal decongestants, nasal obstruction treatment, patterns, saudi arabia

## Abstract

Background: Nasal congestion, a common symptom of various upper respiratory conditions, is often treated with nasal decongestants, which are effective vasoconstrictors used to relieve blockage in conditions like allergic rhinitis, rhinosinusitis, and nasal polyps. However, frequent use of these decongestants can lead to both local and systemic side effects.

Methods: This cross-sectional study used an online, self-administered questionnaire to collect data from our population as regard on the inclusion and exclusion criteria. Inclusion criteria encompassed all consenting individuals aged 10 years and older residing in the Al-Qassim region. Respondents who failed to complete the questionnaire or were not residents of the region were excluded. The structured questionnaire included the participants' demographic information, usage patterns, and knowledge of nasal decongestants. Participants' information was gathered via an online survey that had already been created. Data collected were analyzed using SPSS version 27 (IBM Corp., Armonk, NY, US).

Results: Of the 421 participants, 295 (70.1%) reported prior use of nasal decongestants, with 195 (66.1%) using them for less than five days and 144 (48.8%) reporting twice-daily use. The most common reasons for use were nasal obstruction (128 (43.3%)), rhinosinusitis (68 (23.1%)), and common cold (61 (20.7%)). While 166 (56.3%) used decongestants based on a physician’s recommendation, 120 (40.7%) received no guidance. Pharmacies were the primary source of purchase (211 (71.5%)), and 159 (54.0%) reported using one bottle per month or less. Symptom relief was reported by 265 (89.8%), whereas 33 (11.2%) experienced physician-diagnosed complications. Regarding knowledge, 279 (66.3%) were aware that nasal decongestants relieve congestion, but only 83 (19.7%) recognized the risk of rebound congestion. Although 285 (67.7%) understood that these medications treat symptoms rather than causes, misconceptions regarding pediatric safety and contraindications were prevalent. Overall, 251 (59.6%) had moderate knowledge. A statistically significant association was observed between monthly income and knowledge level (p = 0.018), indicating poorer awareness among lower-income individuals.

Conclusion: Despite noting a high prevalence of the use of nasal decongestants in the Al-Qassim region, the study also reveals a lack of public awareness regarding their safe use, potential adverse effects, and appropriate usage, particularly among lower-income groups. These findings highlight the need for targeted educational interventions to promote safe and effective use.

## Introduction

Nasal congestion, often characterized by a sense of blockage, fullness, or impaired airflow, is a hallmark symptom of several prevalent upper respiratory conditions, such as allergic rhinitis, acute and chronic rhinosinusitis, and nasal polyposis. This condition significantly compromises patients' quality of life, notably through disturbances in sleep and limitations in daily activities [1,2].

Nasal decongestants are frequently utilized therapeutic agents for the treatment of various otorhinolaryngology conditions, for example, allergic rhinitis. Vasoconstrictive drugs alleviate nasal congestion by constricting the dilated blood vessels in the nasal mucosa, thereby reducing swelling and opening the nasal airways to improve airflow. They are available in both systemic and topical forms, such as drops and sprays, and are commonly used to relieve nasal obstruction, facilitate airway patency, reduce nasal secretions, and alleviate discomfort associated with various conditions. For optimal safety and efficacy, it is essential that patients are thoroughly educated about the formulation, active ingredients, and proper dosage [3].

This pharmacological class has been associated with the onset of nasal congestion shortly following administration, a condition referred to as rebound congestion or rhinitis medicamentosa, which is postulated to result from the stimulation of beta-adrenergic receptors. Furthermore, chronic use of topical decongestants may induce pharmacological tolerance, potentially due to receptor downregulation, thereby diminishing therapeutic efficacy over time. The development of rebound congestion and tolerance may prompt patients to increase their use of the medication, which can raise the risk of side effects and result in rhinitis medicamentosa; therefore, the use of these medications should be limited to a maximum of three to five consecutive days [4-6].

Prolonged use of nasal decongestants can increase the risk of toxic effects, including central nervous system symptoms ranging from sedation to stimulation (e.g., insomnia and hallucinations), as well as overdose symptoms such as headache, dizziness, visual disturbances, blood pressure fluctuations, and nausea [7]. Therefore, it is crucial for patients to have a clear understanding of their medications, including their names, purposes, dosing schedules, potential side effects, and any special instructions. This knowledge helps to reduce prescription errors, improve adherence, and enhance patient satisfaction [8,9].

Currently, only one study has investigated public knowledge and utilization patterns of nasal decongestants within the Al-Qassim region [10]. That study, published in 2022, was geographically confined to the city of Unaizah, thereby constraining the extent to which its findings can be generalized to the wider regional population. Given the variation in demographics and access to healthcare across Al-Qassim, there remains a clear gap in assessing awareness at the regional level. Our study aims to address this gap by evaluating knowledge and utilization patterns of nasal decongestants among the general population across the wider Al-Qassim region.

## Materials and methods

Study design

A community-based cross-sectional study was conducted among the general population in the Al-Qassim region of Saudi Arabia over a three-month period, from February to April 2025.

Study population

The target population included individuals aged 10 years and older who were residents of the Al-Qassim region, Saudi Arabia.

Sample size calculation

The required sample size was calculated using the Raosoft Sample Size Calculator (Raosoft, Inc., Seattle, WA, USA). Based on the total population of the Al-Qassim region, a reported nasal decongestant use prevalence of 45.1% in Saudi Arabia [11], a 95% confidence level, and a 5% margin of error, the minimum required sample size was determined to be 385 participants.

Survey development and validation

The study employed a modified, pre-validated self-administered questionnaire adapted from a previous study to align with the current research objectives [8]. The survey was available in both Arabic and English and comprised three main sections. The first section collected sociodemographic information, including region, age, gender, nationality, marital status, education level, monthly income, smoking status, daily cigarette consumption (if applicable), and employment status. The second section focused on nasal decongestant usage patterns, covering aspects such as prior use, duration and frequency of use, types used, reasons for use, concurrent medications, source of recommendation, whether guidance was provided, purchasing sources, monthly usage, symptom improvement, and any diagnosed complications. The third section assessed participants’ knowledge regarding nasal decongestants, addressing their effectiveness in relieving congestion, the risk of rebound congestion from prolonged use, appropriate usage, safety in children, risks associated with sharing, potential side effects, optimal application methods, and known contraindications.

Data collection procedures

Data were collected from a convenience sample of 421 participants via an electronically self-administered questionnaire. The survey was distributed through social media platforms, including WhatsApp (Meta Platforms, Inc., Menlo Park, CA, US) and X (formerly Twitter) (X Corp., San Francisco, CA, US), targeting residents of the Al-Qassim region.

Ethical considerations

Ethical approval was obtained from the Committee of Research Ethics at Qassim University (Approval No. 25-29-15, dated March 13, 2025). Participation was voluntary, and all responses were kept anonymous and confidential.

Statistical analysis

All data were analyzed using SPSS version 27 (IBM Corp., Armonk, NY, US). Descriptive statistics, including frequencies and percentages, were used to summarize demographic characteristics, usage patterns, and knowledge levels. Associations between categorical variables were examined using the Chi-squared test.

## Results

**Figure 1:**
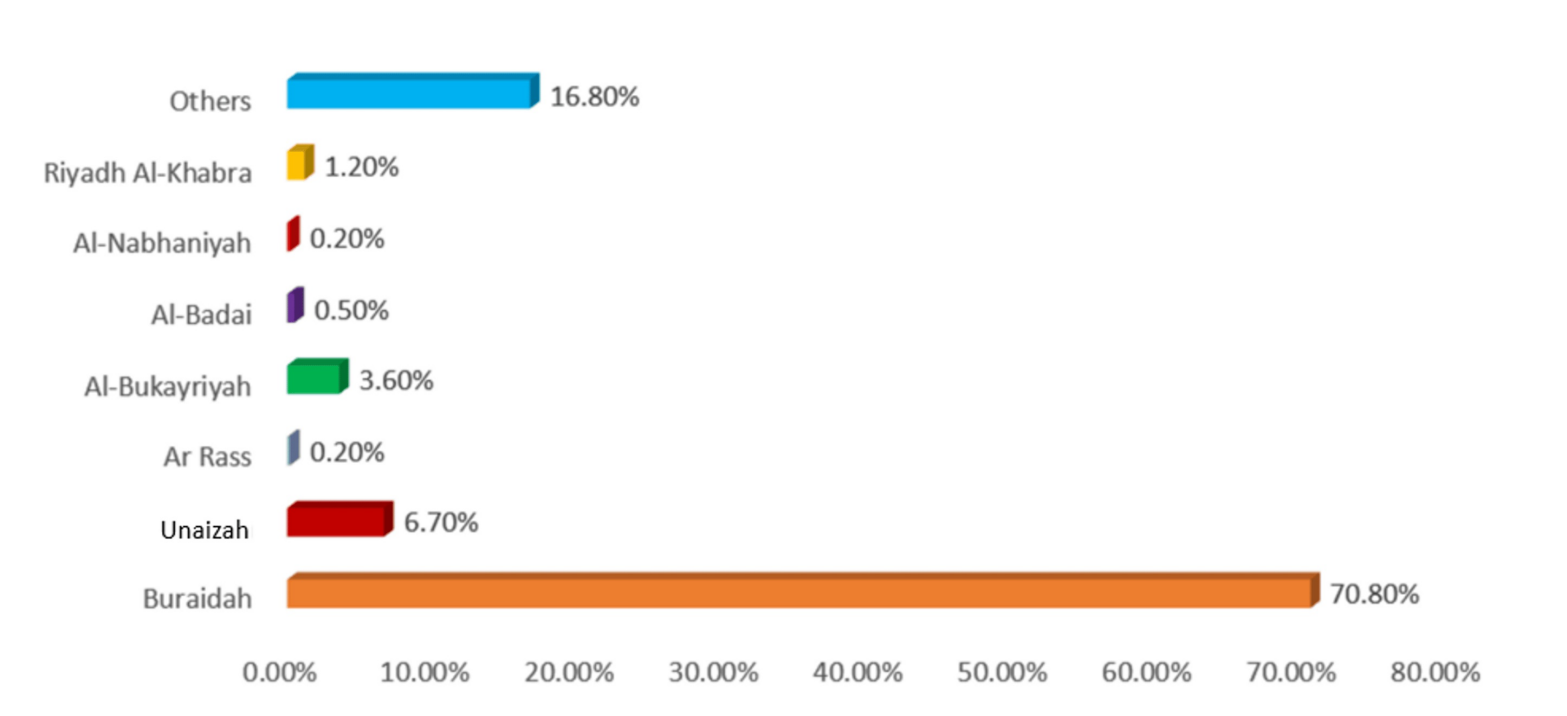
City of Residence

The study included 421 participants with a mean age of 36.79 ± 14.06 years. The majority were male (285 (67.7%)), Saudi nationals (394 (93.6%)), and married (232 (55.1%)). Most participants held a university degree or higher (329 (78.1%)). Regarding income, 161 (38.2%) reported earning less than 5,000 Riyal per month. A large proportion were non-smokers (354 (84.1%)), and government employees (140 (33.3%)) and students (130 (30.9%)) made up the largest employment groups (Table 1). Additionally, 70.8% lived in Buraidah, followed by Unaizah (6.7%), Al-Bukayriyah (3.6%), Riyadh Al-Khabra (1.2%), Al-Badai (0.5%), Ar Rass (0.2%), Al-Nabhaniyah (0.2%), and other areas of Al-Qassim (16.8%) (Figure 1).

**Table 1:** Social-Demographic Characteristics of the Participants (N = 421) Data have been presented as n, %, and mean ± SD.

Variables	Category	n (%)
Age	Mean ± SD	36.79 ± 14.06
Gender	Female	136 (32.3%)
	Male	285 (67.7%)
Nationality	Non-Saudi	27 (6.4%)
	Saudi	394 (93.6%)
Marital status	Divorced	9 (2.1%)
	Married	232 (55.1%)
	Single	180 (42.8%)
Education level	Below secondary	7 (1.7%)
	Secondary	85 (20.2%)
	University and above	329 (78.1%)
Monthly income in Riyal	Less than 5,000 Riyal	161 (38.2%)
	5,000-15,000 Riyal	138 (32.8%)
	More than 15,000 Riyal	122 (29.0%)
Smoking status	Current smoker	51 (12.1%)
	Ex-smoker	16 (3.8%)
	Non-smoker	354 (84.1%)
How many packets/day	Less than 1	39 (9.3%)
	1-2	23 (5.5%)
	I don’t smoke	359 (85.2%)
Work section	Government sector	140 (33.3%)
	Military sector	8 (1.9%)
	Private sector	68 (16.1%)
	Student	130 (30.9%)
	Retired	40 (9.5%)
	Unemployed	35 (8.3%)

According to Table 2, a majority of respondents (295 (70.1%)) reported previous use of nasal decongestants, while 126 (29.9%) had not. Among users, the most common duration was less than five days (195 (66.1%)), followed by 5-15 days (48 (16.3%)). Regarding frequency of daily use, 144 (48.8%) used decongestants twice per day, 56 (19.0%) once per day, and 65 (22.0%) only when symptomatic. The main reasons for decongestant use were nasal obstruction (128 (43.3%)), rhinosinusitis (68 (23.1%)), and common cold (61 (20.7%)).

**Table 2:** Prevalence/Pattern of Nasal Decongestant Use Data have been presented as n, %, and mean ± SD.

Variables	Category	n (%)
Did you previously use nasal decongestants?	No	126 (29.9%)
	Yes	295 (70.1%)
If yes, what is the duration of using decongestants?	Less than 5 days	195 (66.1%)
	5-15 days	48 (16.3%)
	16-30 days	6 (2.0%)
	2-6 months	12 (4.1%)
	7-12 months	3 (1.0%)
	More than 1 year	31 (10.5%)
What is the frequency of using decongestants/day?	1 time/day	56 (19.0%)
	2	144 (48.8%)
	3	25 (8.5%)
	4	4 (1.4%)
	5 or more/day	1 (0.3%)
	Only with symptoms	65 (22.0%)
What are the causes of using nasal decongestants?	Nasal obstruction	128 (43.3%)
	Common cold	61 (20.7%)
	Itching	2 (0.7%)
	Sneezing	12 (4.1%)
	Rhinosinusitis	68 (23.1%)
	Allergic rhinitis	18 (6.1%)
	Hay fever	3 (1.0%)
	Others (1 case runny nose, 1 case middle ear, and 1 case before and after adenoid surgery)	3 (1.0%)
What are other medications used with nasal decongestants? (multiple choice responses)	None	135 (45.8%)
	Oral antihistamine	117 (39.7%)
	Paracetamol	76 (25.8%)
	Others (Lucotrins, salt steam, Lorin, betaseric, Fast 180, Ventolin, Zolax and Airfast, systemic decongestants, and Panadol)	9 (3.1%)
Who recommended nasal decongestants for you?	Physician	166 (56.3%)
	Pharmacist	40 (13.6%)
	Family	24 (8.1%)
	Friends	7 (2.4%)
	Myself	55 (18.6%)
	Internet	3 (1.0%)
Did any person offer advice on how to use a nasal decongestant?	No	120 (40.7%)
	Yes	175 (59.3%)
What is the main site for purchasing the nasal decongestants?	Hospital	43 (14.6%)
	Primary healthcare center	41 (13.9%)
	Pharmacy	211 (71.5%)
How many bottles are used/month?	One	159 (54.0%)
	Two	11 (3.7%)
	Three	3 (1.0%)
	Four/more	6 (2.0%)
	Less than that	116 (39.3%)
Did your symptoms improve with the use of a decongestant?	No	30 (10.2%)
	Yes	265 (89.8%)
Were you diagnosed by a physician for any complications due to nasal decongestants?	No	262 (88.8%)
	Yes	33 (11.2%)

Among participants, 117 (39.7%) reported using oral antihistamines concomitantly with nasal decongestants, while 76 (25.8%) used paracetamol, and nine (3.1%) used other agents (e.g., leukotriene receptor antagonists, loratadine, fexofenadine, salbutamol, montelukast, saline inhalation, betahistine, and oral decongestants).

As for recommendations, 166 (56.3%) received decongestants from a physician, 55 (18.6%) were self-recommended, 40 (13.6%) were recommended by a pharmacist, 24 (8.1%) by family, seven (2.4%) by friends, and three (1.0%) from the internet. Usage guidance was reported by 175 (59.3%), while 120 (40.7%) had received no instructions. Most participants purchased decongestants from pharmacies (211 (71.5%)), followed by hospitals (43 (14.6%)) and primary healthcare centers (41 (13.9%)). Monthly consumption showed that 159 (54.0%) used one bottle, 116 (39.3%) used less than one, 11 (3.7%) used two, six (2.0%) used four or more, and three (1.0%) used three bottles.

The majority (265 (89.8%)) reported symptom improvement with decongestant use, while 30 (10.2%) did not. Regarding complications, 262 (88.8%) had none diagnosed by a physician, while 33 (11.2%) reported complications.

**Figure 2:**
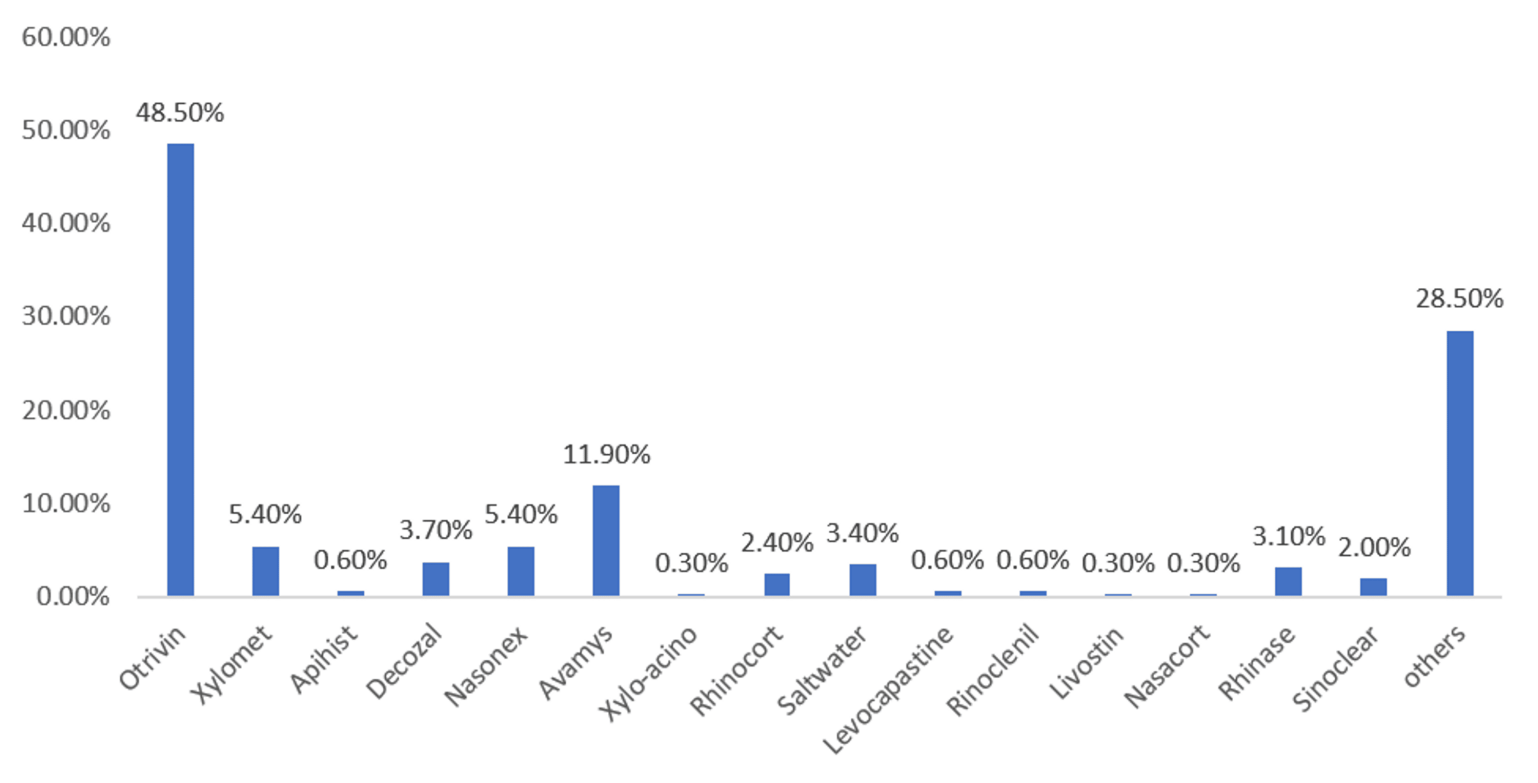
Pattern of Nasal Decongestant Use and Other Medications Used for Nasal Symptom Relief

The bar chart in Figure 2 illustrates the distribution of nasal spray usage among respondents. Xylometazoline (48.5%) was the most commonly used nasal decongestant, followed by fluticasone furoate (11.9%). A notable proportion of participants also reported using fluticasone furoate, a corticosteroid spray, likely due to its use in allergic rhinitis management.

Table 3 summarizes participants' awareness regarding nasal decongestants. While 279 (66.3%) correctly identified that nasal decongestants relieve symptoms of congestion and rhinitis, only 83 (19.7%) were aware of the risk of rebound congestion with use beyond five days. Additionally, 285 (67.7%) understood that these medications offer symptomatic relief only, yet 246 (58.4%) were unsure about their safety in children.

**Table 3:** Knowledge of Nasal Decongestants (N = 421) Data have been presented as n and %.

Variables	Category	n (%)
Do nasal decongestants relieve the symptoms of congestion and rhinitis associated with the common cold?	I don’t know	106 (25.1%)
	No	36 (8.6%)
	Yes	279 (66.3%)
Do nasal decongestants cause rebound congestion when used for more than 5 days?	I don’t know	266 (63.2%)
	No	72 (17.1%)
	Yes	83 (19.7%)
Are nasal decongestants used for symptomatic treatment only?	I don’t know	100 (23.7%)
	No	36 (8.6%)
	Yes	285 (67.7%)
Are nasal decongestants safe to use in children?	I don’t know	246 (58.4%)
	No	73 (17.4%)
	Yes	102 (24.2%)
Do you think the topical agents are not effective because they are weakly absorbed by the systemic circulation?	I don’t know	254 (60.3%)
	No	101 (24.0%)
	Yes	66 (15.7%)
Do you think patients can share a bottle of nasal decongestants with other people because there is no risk of infection?	I don’t know	100 (23.8%)
	No	286 (67.9%)
	Yes	35 (8.3%)
Are nasal decongestants among the major classes of drugs responsible for poisoning and death of children under five years of age?	I don’t know	307 (72.9%)
	No	73 (17.3%)
	Yes	41 (9.7%)
What is/are the side effects of prolonged use of topical nasal decongestants?	Nasal dryness	135 (32.0%)
	Ulceration of the nasal mucosa	74 (17.6%)
	I don’t know	212 (50.4%)
What is the best position when using topical nasal decongestants?	To tilt one’s head forward, dripping drops in the amount indicated	247 (58.7%)
	A person can put the drops in any position with no special position	69 (16.4%)
	I don’t know	105 (24.9%)
Does nasal instillation of saline an adjuvant for nasal decongestants and effective?	I don’t know	133 (31.6%)
	No	43 (10.2%)
	Yes	245 (58.2%)
What are the contraindications of the systemic form of nasal decongestants?	Hypertension	73 (16.3%)
	Hyperthyroidism	4 (1.0%)
	Seizure	1 (0.2%)
	Glaucoma	5 (1.2%)
	Ischemic heart disease	6 (1.4%)
	Prostatic disease	4 (1.0%)
	I don’t know	328 (77.9%)

**Figure 3:**
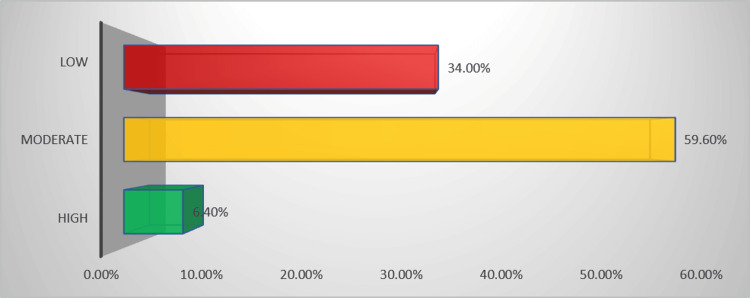
Knowledge Level

Misconceptions were notable: 254 (60.3%) did not know whether poor systemic absorption affects topical agents’ efficacy, and only 35 (8.3%) incorrectly believed sharing bottles poses no infection risk. Awareness of toxicity was also low, with 307 (72.9%) unaware that nasal decongestants can be harmful to children under five. When asked about the adverse effects of prolonged use, 135 (32.0%) reported nasal dryness and 74 (17.6%) mucosal ulceration, while 212 (50.4%) were unsure. Regarding administration, 247 (58.7%) identified the correct head-tilt technique. A total of 245 (58.2%) agreed that saline is an effective adjuvant. Knowledge of contraindications for systemic nasal decongestants was poor; 328 (77.9%) were unaware, with hypertension noted by only 73 (16.3%). Overall, most participants-251 (59.6%)-demonstrated a moderate level of knowledge (Figure 3).

Table 4 shows a statistically significant association between participants’ monthly income and their knowledge level of nasal decongestants (p = 0.018). A higher proportion of participants with high knowledge levels reported earning more than 15,000 SR (14, 51.9%), compared to those with moderate (75, 29.9%) and low (33, 23.0%) knowledge. Conversely, a greater percentage of those with low knowledge had an income below 5,000 SR (61, 42.7%). No statistically significant associations were observed with other sociodemographic variables such as gender, nationality, education, or smoking status.

**Table 4:** Association Between Social-Demographic Factors and Knowledge Level of Nasal Decongestants Data have been presented as n and %. A p-value less than 0.05 was significant.

Variables	Category	High n (%)	Moderate n (%)	Low n (%)	p-value
Gender	Female	10 (37.0%)	84 (33.5%)	42 (29.4%)	0.608
	Male	17 (63.0%)	167 (66.5%)	101 (70.6%)	
Nationality	Non-Saudi	2 (7.4%)	19 (7.6%)	6 (4.2%)	0.412
	Saudi	25 (92.6%)	232 (92.4%)	137 (95.8%)	
Marital status	Divorced	0 (0.0%)	5 (2.0%)	4 (2.8%)	0.159
	Married	11 (40.7%)	149 (59.4%)	72 (50.3%)	
	Single	16 (59.3%)	97 (38.6%)	67 (46.9%)	
Education level	Below secondary	1 (3.7%)	5 (2.0%)	1 (0.7%)	0.657
	Secondary	5 (18.5%)	54 (21.5%)	26 (18.2%)	
	University and above	21 (77.8%)	192 (76.5%)	116 (81.1%)	
Monthly income	Less than 5,000 SR	10 (37.0%)	90 (35.9%)	61 (42.7%)	0.018
	5,000-15,000 SR	3 (11.1%)	86 (34.3%)	49 (34.3%)	
	More than 15,000 SR	14 (51.9%)	75 (29.9%)	33 (23.0%)	
Smoking status	Current smoker	5 (18.5%)	32 (12.7%)	14 (9.8%)	0.479
	Ex-smoker	2 (7.4%)	10 (4.0%)	4 (2.8%)	
	Non-smoker	20 (74.1%)	209 (83.3%)	125 (87.4%)	
How many packets/day	Less than 1	4 (14.8%)	25 (10.0%)	10 (7.0%)	0.560
	1-2	2 (7.4%)	15 (6.0%)	6 (4.2%)	
	I don’t smoke	21 (77.8%)	211 (84.0%)	127 (88.8%)	
Work section	Government sector	9 (33.3%)	88 (35.1%)	43 (30.1%)	0.062
	Military sector	2 (7.4%)	3 (1.2%)	3 (2.1%)	
	Private sector	3 (11.2%)	48 (19.1%)	17 (11.9%)	
	Student	12 (44.4%)	71 (28.3%)	47 (32.9%)	
	Retired	0 (0.0%)	21 (8.3%)	19 (13.2%)	
	Unemployed	1 (3.7%)	20 (8.0%)	14 (9.8%)	

## Discussion

Nasal congestion is a common symptom of upper respiratory disorders, often alleviated by nasal decongestants [12]. However, prolonged use can cause negative effects [13]. This study aims to assess the prevalence and the related knowledge of nasal decongestants among the general population in the Al-Qassim region, Saudi Arabia. There was a general knowledge gap in the population, with the majority of participants demonstrating a moderate level of knowledge regarding nasal decongestants (59.6%), followed by low knowledge (34.0%), and only a small proportion showing high knowledge (6.4%). This indicates the need for improved public education and awareness about the safe and appropriate use of nasal decongestants. Our finding aligns with a study conducted among the Saudi general population by Almutairi et al. who reported that although 64.4% of participants had previously used nasal decongestants, only 16.6% were aware of their side effects, 25.2% knew the medically recommended duration of use, 21.3% had heard of nasal congestion addiction, and 21.6% were aware of medications that may cause nasal congestion [10].

In this study, nasal decongestant use was found to be highly prevalent among the general population in the Al-Qassim region, with 70.1% of participants reporting prior use. Otrivin (48.5%) was identified as the most commonly used spray. Similarly, a study conducted among the general population in Saudi Arabia reported a prevalence of 76.6% [11]. The high usage observed in both studies is likely due to the easy accessibility of these sprays, their rapid symptomatic relief, and the widespread public perception of their effectiveness in managing nasal congestion.

Among these users, 66.1% indicated they used nasal decongestants for less than five days, while only 10.5% reported usage extending beyond one year. Notably, 48.8% used the decongestants twice daily, reflecting a pattern of frequent short-term use. These findings are in contrast with those of Alharthi et al., where only 45.1% of participants had ever used nasal decongestants. In terms of usage duration, their study also found that 70.8% used the medication for less than five days, and 13.5% used it for 5-15 days [11]. The discrepancy in overall prevalence between the two studies may be attributed to differences in target populations and regional characteristics. While the present study focused specifically on residents in the Al-Qassim region, Alharthi et al. may have surveyed a broader or different demographic group with varying health-seeking behaviors, levels of health literacy, or access to healthcare services.

In regard to daily consumption patterns, while a considerable number of participants reported using nasal decongestants twice daily, smaller percentages indicated more frequent use, such as three or more times per day, suggesting that intensive or prolonged use was less common in the population. Nasal obstruction (43.3%), rhinosinusitis (23.1%), and the common cold (20.7%) were the most prevalent reasons for nasal decongestant administration. A study by Alkalash et al. revealed that nasal obstruction was the leading cause (65.9%), followed by the common cold (40.1%) and rhinosinusitis (25.2%) [8]. The slight variation may be due to differences in population.

The main source (56.3%) of information regarding nasal decongestants came from doctors, followed by pharmacists (13.6%), self-sourcing (18.6%), and advice from family and friends. However, almost half (40.7%) of the participants did not receive information on how to use nasal decongestants, despite the fact that the majority (59.3%) did. Furthermore, most users (54.0%) reported using one bottle or less per month, with purchases from pharmacies accounting for 71.5%. While 11.2% of patients had complications diagnosed by a physician, the majority (89.8%) reported a reduction in symptoms following the use of nasal decongestants. The results show that nasal decongestants are highly used, mostly under medical guidance, with generally favorable results but a significant percentage of protracted use and sporadic side effects. Similar to this finding, a study by Rajasekaran et al. revealed that the majority of users used nasal decongestants after a doctor's prescription; however, only 14.3% of users adhered to the guidelines. The study also noted that long-term use reduced symptoms, but there were adverse effects noted among the group [14]. Salama et al. further reported that the main reasons for using nasal decongestants included nasal blockage and the common cold. The study also noted that side effects were common among the users. Similarly, as noted in this study, the main source of information on usage was doctors and pharmacists [15].

This study found that while 66.3% of participants were aware that nasal decongestants relieve symptoms like congestion and rhinitis, only 19.7% recognized the risk of rebound congestion from using nasal decongestants for more than five days. A majority (67.7%) correctly understood that nasal decongestants address symptoms rather than the underlying condition. However, only 24.2% believed nasal decongestants were safe for children, and 15.7% thought topical therapies were ineffective due to limited systemic absorption-reflecting misconceptions about their safety and efficacy. The lack of awareness regarding rebound congestion and the inappropriate use of nasal decongestants in children raise significant clinical concerns. Unsupervised or prolonged use may lead to complications such as rhinitis medicamentosa, mucosal damage, and dependence on topical agents. Additionally, the misconception that these medications are harmless for pediatric use may increase the risk of toxicity, especially considering the narrow safety margin in younger age groups. These findings highlight the need for primary care physicians and pharmacists to proactively educate patients on proper usage, duration limits, and age-specific contraindications.

Encouragingly, 67.9% knew that nasal decongestants should not be shared due to infection risks, and 58.7% were aware that the correct application involves tilting the head forward. Additionally, 58.2% supported saline nasal instillation as a helpful adjunct therapy. Despite this, understanding of contraindications was poor; 77.9% were unaware of any, even though 16.3% had hypertension. Awareness of side effects was also limited: only 32.0% reported dry nose and 17.6% acknowledged nasal mucosal ulcers. These findings align with Almutairi et al., where only 16.6% were aware of adverse effects, 25.2% knew the recommended duration of use, and 21.3% had heard of nasal congestion addiction [10]. Similarly, Alarfaj et al. found that while 84.3% of users experienced side effects, just 29.3% were aware of long-term risks, and 39.5% understood why symptoms returned after stopping nasal decongestants [16].

Finally, this study found a statistically significant association between the participants’ monthly income and knowledge level of nasal decongestants (p = 0.018). More than half of the participants (51.9%) who had a monthly income of 15,000 SR had a high knowledge level of nasal decongestants. Conversely, almost half of the participants (42.7%) who earned less than 5,000 SR had a low knowledge level of nasal decongestants. Unlike the association found in this study, a study by Alkalash et al. reported that higher knowledge scores were linked to both being a student and being female [8]. However, the diversity in the association may be explained by the target group. This call for a comparison study focused on a similar niche.

There are some limitations to this study. Primarily, the study cohort was drawn from a single geographical region, which may limit the generalizability of our findings. Furthermore, this study survey was administered electronically, which limits its use to an educated group of participants. Also, there is a lack of comparable international studies on nasal decongestant use, which limits our ability to contextualize the findings on a global scale. The use of a convenience sampling method is linked to the maldistribution of the target sample, although this could be avoided by employing the cluster sampling strategy. However, despite the previously mentioned limitations, this study has insightful findings, which may help policymakers and healthcare practitioners to construct health education interventions and launch awareness campaigns based on the revealed gaps in public understanding regarding nasal decongestants. Furthermore, this study can help other researchers to carry out a series of new assessments into various sorts of over-the-counter drugs, especially in remote areas with minimal healthcare services.

## Conclusions

This study demonstrates that nasal decongestants are widely used among the general population in the Al-Qassim region, with most users relying on medical guidance and experiencing symptom relief. However, substantial gaps exist in public knowledge regarding safe use, potential side effects, and the appropriate duration of treatment. While the majority demonstrated moderate awareness, critical misconceptions such as the risks of prolonged use and safety in children are common. There was a significant association between higher income and better knowledge levels. To address these concerns, we recommend implementing public awareness campaigns, incorporating medication safety counseling into routine pharmacy and clinical encounters, and including nasal decongestant education in health literacy programs. Additionally, since pharmacists play a vital role in educating the public on the safe and appropriate use of nasal decongestants, it is important to consult before initiating use, which can help individuals understand the correct duration, avoid complications such as rebound congestion, and ensure that the medication is suitable given their health status and concurrent medications. In order to identify the most effective methods for bridging these knowledge gaps, it is recommended that future research concentrate on assessing the efficacy of educational interventions across various demographic groups.

## Appendices

Questionnaire

a. Consent to Participate

Do you agree to participate in this study?

A. Yes

B. No

b. Sociodemographic Information

Region:

A. Buraidah

B. Unaizah

C. Ar Rass

D. Al-Bukayriyah

E. Al-Badai

F. Al-Nabhaniyah

G. Riyadh Al-Khabra

H. Others

Age: ________

Gender:

A. Male

B. Female

Nationality:

A. Saudi

B. Non-Saudi

Marital Status:

A. Single

B. Married

C. Divorced

D. Widow

Educational Level:

A. Below secondary

B. Secondary

C. University or above

Monthly Income (SAR):

A. Less than 5,000

B. 5,000-15,000

C. More than 15,000

Smoking Status:

A. Current smoker

B. Ex-smoker

C. Non-smoker

If a smoker, how many packets/day?

A. Less than 1 packet/day

B. 1-2 packets/day

C. More than 2 packets/day

D. I don’t smoke

Employment Sector:

A. Military sector

B. Private sector

C. Government sector

D. Student

E. Non-worker

F. Retired

c. Nasal Decongestant Usage Pattern

Have you previously used nasal decongestants?

A. Yes

B. No

Duration of use:

A. Less than 5 days

B. 5-15 days

C. 16-30 days

D. 2-6 months

E. 7-12 months

F. More than 1 year

Frequency of use per day:

A. 1 time/day

B. 2 times/day

C. 3 times/day

D. 4 times/day

E. 5 or more times/day

F. Only with symptoms

Types of nasal decongestants used (select all that apply):

A. Otrivin

B. Xylomet

C. Apihist

D. Decozal

E. Nasonex

F. Avamys

G. Xylo-acino

H. Otrizethic

I. Rhinocort

J. Saltwater

K. Levocapastine

L. Rinoclenil

M. Livostin

N. Nasacort

O. Nasonex (duplicate, consider removing)

P. Rhinase

Q. Rinoclenil 100

R. Sinoclear

S. Sterimar

T. Other (please specify): __________

Reasons for using nasal decongestants (select all that apply):

A. Nasal obstruction

B. Common cold

C. Itching

D. Sneezing

E. Rhinosinusitis

F. Allergic rhinitis

G. Hay fever

H. Other (please specify): __________

Other medications used concurrently (select all that apply):

A. None

B. Oral antihistamine

C. Paracetamol

D. Other (please specify): __________

Who recommended nasal decongestants?

A. Physician

B. Pharmacist

C. Family

D. Friends

E. Myself

F. Internet

Were you given advice on how to use nasal decongestants?

A. Yes

B. No

Where do you mainly purchase nasal decongestants?

A. Pharmacy

B. Primary healthcare center

C. Hospital

How many bottles do you use per month?

A. One

B. Two

C. Three

D. Four or more

Did your symptoms improve with nasal decongestant use?

A. Yes

B. No

Have you been diagnosed by a physician with any complications related to nasal decongestant use?

A. Yes

B. No

If yes, please specify the complications: _______________

d. Knowledge About Nasal Decongestants

Do nasal decongestants relieve congestion and rhinitis symptoms related to the common cold?

A. Yes

B. No

C. I don’t know

Can nasal decongestants cause rebound congestion if used for more than 5 days?

A. Yes

B. No

C. I don’t know

Are nasal decongestants used for symptomatic treatment only?

A. Yes

B. No

C. I don’t know

Are nasal decongestants safe to use in children?

A. Yes

B. No

C. I don’t know

Are topical nasal agents ineffective due to weak systemic absorption?

A. Yes

B. No

C. I don’t know

Is it safe to share a bottle of nasal decongestants with others?

A. Yes

B. No

C. I don’t know

Are nasal decongestants among major drugs responsible for poisoning and death in children under five?

A. Yes

B. No

C. I don’t know

What are the side effects of prolonged use of topical nasal decongestants? (select all that apply)

A. Nasal dryness

B. Ulceration of the nasal mucosa

C. I don’t know

What is the best position for using topical nasal decongestants?

A. Tilt head forward, drip drops as indicated

B. Drops can be applied in any position

C. I don’t know

Is nasal saline instillation an effective adjuvant treatment?

A. Yes

B. No

C. I don’t know

What are contraindications of systemic nasal decongestants? (select all that apply)

A. Hypertension

B. Hyperthyroidism

C. Seizure

D. Glaucoma

E. Difficulty in urination

F. Ischemic heart disease

G. Prostatic diseases

H. I do not know
